# Axonemal Symmetry Break, a New Ultrastructural Diagnostic Tool for Primary Ciliary Dyskinesia?

**DOI:** 10.3390/diagnostics12010129

**Published:** 2022-01-06

**Authors:** Rosana Blanco-Máñez, Miguel Armengot-Carceller, Teresa Jaijo, Francisco Vera-Sempere

**Affiliations:** 1 Pathology Department, La Fe Polytechnic and University Hospital, 46020 Valencia, Spain; 2 Molecular, Cellularand Genomic Biomedicine Group, IIS La Fe, 46020 Valencia, Spain; miguel.armengot@gmail.com (M.A.-C.); tjaijo@gmail.com (T.J.); 3 Surgery Department, University of Valencia, 46010 Valencia, Spain; 4 CIBER of Respiratory Diseases (CIBERES), Carlos III Health Institute, Ministerio de Ciencia e Innovación, 28029 Madrid, Spain; 5 ENT Department, La Fe Polytechnic and University Hospital, 46020 Valencia, Spain; 6 Genetics Department, La Fe Polytechnic and University Hospital, 46020 Valencia, Spain; 7 CIBER of Rare Diseases (CIBERES), Carlos III Health Institute, Ministerio de Ciencia e Innovación, 28029 Madrid, Spain; 8 Pathology Department, University of Valencia, 46010 Valencia, Spain; fco.jose.vera@uv.es

**Keywords:** primary ciliary dyskinesia, diagnosis, ciliary axoneme, transmission electron microscopy

## Abstract

Diagnosis testing for primary ciliary dyskinesia (PCD) requires a combination of investigations that includes study of ciliary beat pattern by high-speed video-microscopy, genetic testing and assessment of the ciliary ultrastructure by transmission electron microscopy (TEM). Historically, TEM was considered to be the “gold standard” for the diagnosis of PCD. However, with the advances in molecular genetic techniques, an increasing number of PCD variants show normal ultrastructure and cannot be diagnosed by TEM. During ultrastructural assessment of ciliary biopsies of patients with suspicion of PCD, we observed an axonemal defect not previously described that affects peripheral doublets tilting. To further characterize this defect of unknown significance, we studied the ciliary axonemes by TEM from both PCD-confirmed patients and patients with other sino-pulmonary diseases. We detected peripheral doublets tilting in all the PCD patients, without any significant difference in the distribution of ciliary beat pattern or mutated gene. This defect was also present in those patients with normal ultrastructure PCD subtypes. We believe that the performance of axonemal asymmetry analysis would be helpful to enhance diagnosis of PCD.

## 1. Introduction

Primary ciliary dyskinesia (PCD) is a rare genetic disease of motile cilia with an estimated prevalence of 1 per 20,000–40,000 births (ORPHA244). It is associated with abnormal ciliary function and/or structure, resulting in a defective mucociliary clearance [1]. PCD is characterized by chronic upper and lower respiratory tract disease with daily wet cough and rhinorrhea, chronic rhinosinusitis, chronic otitis media and development of bronchiectasis, fertility problems secondary to sperm immotility or impaired ciliary function in the oviduct, and organ laterality defects [2,3].

A precise diagnosis of PCD may be challenging because motile cilia are highly complex structures composed of approximately 250 proteins. A mutational change in any of these proteins leads to different clinical and ciliary phenotypes [4]. Currently, the diagnosis of PCD is based on the presence of a typical clinical phenotype and a combination of technically demanding investigations, including high-speed video-microscopy analysis (HVMA), genetic testing and assessment of the ciliary ultrastructure by transmission electron microscopy (TEM) [5,6].

Since the first ultrastructural description was published in 1976 by Björn Afzelius [7], the ciliary ultrastructural analysis by transmission electron microscopy has played a key role in the PCD diagnostic workflow. However, approximately 30% of PCD cases display apparently normal ciliary ultrastructure [8,9,10].

Concurrently, developments in genomics and molecular medicine are rapidly increasing gene discovery, and nearly 40 genes have been reported to cause PCD [11]. Therefore, a new diagnostic characterization of these patients, including motility and ultrastructural typification, will be necessary.

During the ultrastructural diagnostic evaluation of nasal biopsies of patients suspected of PCD, we observed an axonemal defect not previously described. This defect affected the peripheral doublets orientation of PCD patients, and we believe it could be a new useful tool in ultrastructural diagnosis. We have termed it “axonemal asymmetry”. We proceeded to formally study this defect to test the hypothesis that its presence was predictive of the PCD diagnosis. To determine if axonemal asymmetry was specific to PCD patients, we also studied nasal biopsies from patients with a clinical suspicion for PCD but excluded from the diagnosis, patients with cystic fibrosis, immunodeficiency or idiopathic bronchiectasis.

## 2. Materials and Methods

### 2.1. Ethical Aspects

This was a retrospective, observational, single-center study. A total of 20 patients with a confirmed PCD diagnostic and a control group of 20 patients with a chronic respiratory disease were included in this study. We reviewed the clinical characteristics and diagnostic tests of all the participants.

This study was performed in the Pathology Department of La Fe Polytechnic and University Hospital of Valencia, Spain. Patient recruitment was carried out in the Otorhinolaryngology Department of the same center.

Written informed consent was obtained from all participants or their legal guardians. This study was approved by the Ethics Local Committee of La Fe Hospital (protocol 2021-278-1, 12 May 2021).

### 2.2. Preparation of Nasal Brushings for Analysis by Transmission Electron Microscopy

Samples of nasal curettage were fixed in 2.5% phosphate-buffered glutaraldehyde pH 7.3 for 1 day at 4 °C, washed later twice in the same buffer. The specimens were postfixed in buffered 1% osmium tetroxide for 2 h, washed, dehydrated through a graded series of acetone, cleared in propylene-oxide and embedded in EMbed 812 resin (EMS, Harrisburg, PA, USA)). Semithin sections 200 nm thick, cut with glass knives on a Leica Ultramicrotome EM UC6 and stained with 0.1% toluidine blue, were examined with the light microscope in order to make an overall assessment of the presence of ciliary structures. Ultrathin sections (70 nm thickness) from selected blocks were cut with a diamond knife (DiATOME, Nidau, Switzerland) using the same ultramicrotome, retrieved onto copper grids, double-stained with uranyl acetate and lead citrate and examined at 80 kV with a Hitachi HT7700 transmission electron microscope (Hitachi, Japan).

### 2.3. Diagnostic Assessment of the Ciliary Axonemes

At least 50 cross-sections of cilia from each patient were evaluated to establish the presence of ultrastructural abnormalities. A detailed ultrastructural analysis was undertaken, and all the diagnostic abnormalities were recorded. Results were expressed as a percentage of abnormal axonemes among the total number of analyzed cross-sections. The axonemal defects were classified as a Class 1 defect (hallmark diagnostic defect) or Class 2 defect (indicating a PCD diagnosis with other supporting evidence) in agreement with the BEAT PCD Criteria [12]. For the asymmetry assessment, a preserved axonemal structure is required; hence, patients with microtubular disorganization defects were excluded (Table 1).

### 2.4. Axonemal Asymmetry Assessment

**Figure 1:**
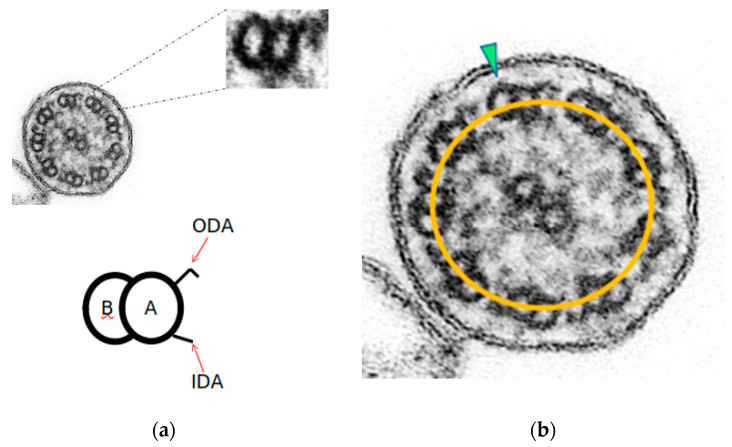
Axonemal symmetry. (**a**) Each microtubule doublet of the ciliary axoneme contains an incomplete B-tubule and a complete A-tubule, with the outer (ODA) and inner (IDA) motor protein dynein arms. (**b**) Ciliary section of (**a**) with preserved axonemal symmetry: both microtubules of each peripheral doublets are aligned with the axonemal circumference (yellow line), and just one doublet is tilted (green arrowhead) (Original magnification ×530,000).

We define the axonemal circumference—or oval—as the line joining the center of all the A-tubules (Figure 1b). In ciliary sections with preserved axonemal structure, the doublets are aligned with their axonemal circumference, and few of them show a slight inclination. These ciliary sections show axonemal symmetry.

**Figure 2:**
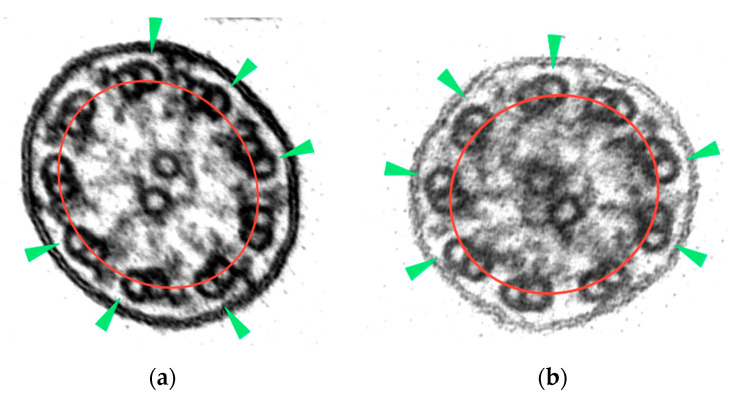
Axonemal symmetry break: (**a**) rotating doublets of ciliary section without apparent axonemal defects; (**b**) rotating doublets of ciliary section with absence of ODAs. B-tubules (green arrowheads) are outside the axonemal circumference (red line) (micrographs, original magnification ×530,000).

In this study, and through a repeated observation method of ciliary sections of non-PCD patients, the axonemal symmetry break was established in those ciliary sections with more than 3 rotated peripheral doublets. To identify this misalignment, the B-tubule should be outside the axonemal circumference (Figure 2).

To assess this symmetry break as an axonemal ultrastructural defect in PCD patients, we retrospectively studied the ciliary biopsies of the PCD patients and the control group of patients excluded from the diagnosis. Axonemal asymmetry (AA) results were expressed as a percentage of axonemes with symmetry breaks among the total number of analyzed cross-sections.

### 2.5. Statistical Analysis

The data were summarized using the mean (SD) and median (1st and 3rd quartiles) in the case of numerical variables and using the absolute frequency (%) in the case of qualitative variables.

## 3. Results

### 3.1. Symmetry Break Analysis

**Figure 3:**
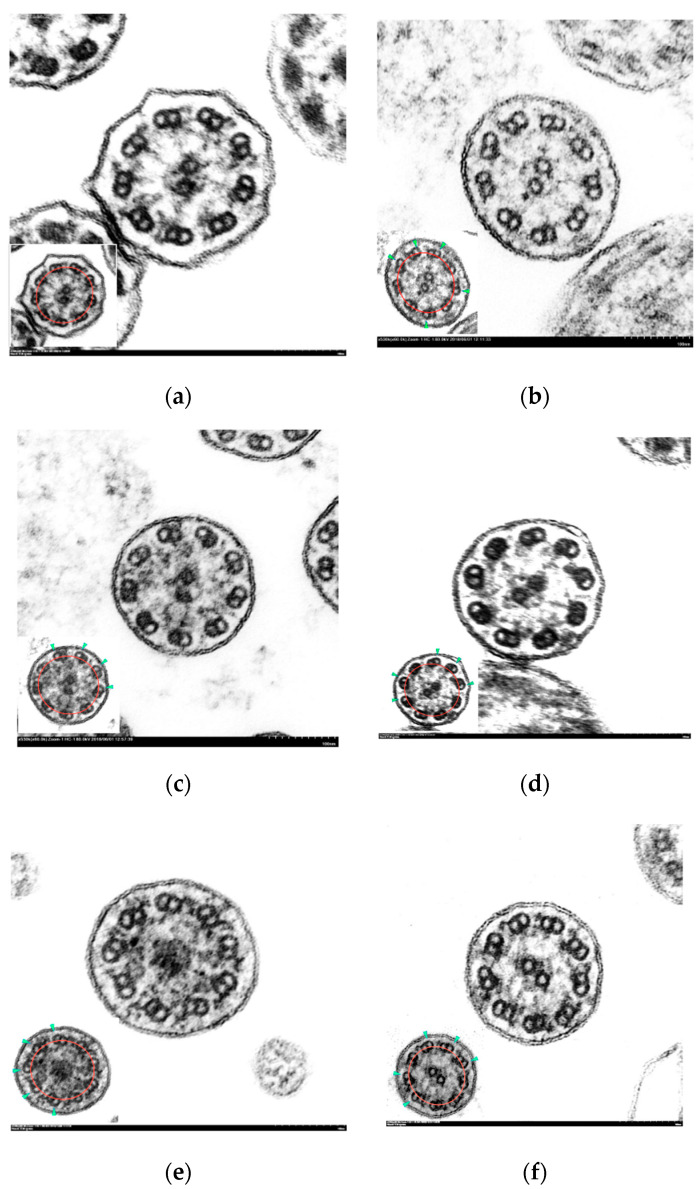
Electron micrographs of transverse sections of cilia with axonemal symmetry break. Inset: ciliary sections with marked axonemal circumference (red line) and tilted doublets (green arrowhead).(**a**) Normal cilium showing conserved ultrastructure and axonemal symmetry; (**b**–**f**) ciliary axonemes with more than 3 tilted peripheral doublets: (**b**) partial absence of outer dynein arms (short arms); (**c**) absence of inner and outer dynein arms; (**d**) complete absence of outer dynein arms; (**e**) central complex defect: absence of central pair; (**f**) ciliary axoneme with normal ultrastructure and axonemal asymmetry. (Micrographs, original magnification ×530,000).

The axonemal asymmetry in PCD patients ranged from 38 to 68% (Table 2). Symmetry breaking was more evident in those ciliary sections with the absence of outer dynein arms (Figure 3b,c), although it was also observed in normal-appearing ultrastructure sections (Figure 3f).

Hence, all the PCD patients in our study showed an AA beyond 38%, regardless of the mutated gene, CCI, ciliary motility or TEM results. We also observed this symmetry break in the ciliary axonemes of the patients with normal ultrastructure (7/20; 35%), even in those with a mutation in *DNAH11* (patient 81) or *RSPH1* (patients 91 and 92), two specific forms of PCD with apparently normal ultrastructure. All the patients showed ultrastructural defects, in diagnostic and non-diagnostic proportions (Table S1), with the exception of patients 91 and 92, in which the AA was the only detected ultrastructural defect.

The data obtained from the control group (Table 3) revealed a minimal axonemal asymmetry proportion of 2%, with a maximal value of 63% in participant 59. Their demographic and clinical characteristics are summarized in Table S2.

Participants 25 and 59 showed AA values within the range observed in PCD patients. Moreover, their detailed ultrastructural analysis revealed non-diagnostic ODA defect rates (16% and 22%, respectively), and higher ODA defect values for the control group (Table S3).

### 3.2. Statistical analysis

The genetic, HSVA and TEM information of PCD patients are summarized in Table S4, and their demographic and clinical characteristics in Table S5.

Our data did not show any significant difference in the distribution of ciliary phenotypes and axonemal asymmetry (Table 4 and Table 5).

## 4. Discussion

This study first reports the observation that axonemal asymmetry is mainly observed in axonemes from patients with PCD and is seen across different PCD phenotypes and genotypes.

Virtually all the PCD patients in our cohort showed impairment of ciliary beat pattern, and all of them showed axonemal symmetry break. In contrast, the control group patients, with a conserved axonemal symmetry, had normal ciliary motility. The exception to this statement was two patients in the control group with raised axonemal asymmetry and moderate absence of outer dynein arms.

We propose several hypotheses for the link between axonemal asymmetry and dysfunctional cilia. It could be pointed to the following subjects: the basal body architecture, some axonemal components, as ODA or nexin link dynein regulator complex proteins (N-DRC) or acquired ciliary defects. Basal bodies give rise to cilia in the last stage of the ciliogenesis process. They provide a ninefold symmetric template on which the ninefold symmetry axonemal structure of the cilium can be built. They are modified centrioles, composed of nine triplet microtubule “blades” arranged in a barrel shape [13]. Nine peripheral doublet microtubules of the ciliary axoneme elongate from A- and B-tubules of the triplet microtubules of the basal body [14], and at the proximal cilium region, peripheral doublets have the characteristic angled-out appearance of basal body triplets. This geometry changes through the axoneme, reducing the doublets angulation but keeping the rotational symmetry of the basal body [15]. Consequently, peripheral doublets show a slight non-pathologic tilt, evidenced in 1984 by Rautiainen et al. in a study of ciliary serial sections [16]. We have termed this conformation as “axonemal symmetry”. Our observations revealed more than three clearly tilted doublets in a significant number of ciliary sections in PCD patients’ samples. A basal body abnormal template could lead to an asymmetric axoneme.

On the other hand, it is estimated that the axoneme must experience considerable distortion during the beat cycle [17]. The N-DRC provides some resistance to microtubule sliding and maintains alignment between outer doublet microtubules during ciliary bending [18]. Some genetic defects encoding the N-DRC have been reported, such as *CCDC164*, *CCDC65* and *GAS8*, and the ultrastructural analysis occasionally shows not well aligned peripheral doublets [19,20,21]. Nonetheless, the main genetic defect in our cohort affects ODA structure, and the cases that revealed the higher axonemal asymmetry values had mutations on *RSPH1* or genes encoding ODA components. It is also important to note that participants 25 and 59, with axonemal asymmetry values in the PCD patients’ range, were the only ones with minor ODA defects. Indeed, each stalk of heavy chains of ODA attached to an A-tubule seems to connect to a consecutive, specific protofilament on the neighboring B-tubule [22]. Considering all the presented data as a whole, peripheral doublets tilting could be due to a novel structural defect localized at ODAs or N-DRC complexes. Further studies would be necessary to elucidate the molecular basis of this finding.

A third explanation of the axonemal symmetry break would be an ultrastructural change secondary to chronic respiratory disease of these patients. Altered ciliogenesis following recurrent infections can lead to ciliary abnormalities—swollen cilia, compound cilia, disorganized axonemes, addition or deletion of peripheral microtubules, or loss of one or both of the central pair of microtubules—but these changes are present in less than 5–10% of the cilia examined and in a localized area [23,24,25]. Thus, the characteristic identifying axonemal asymmetry is shown not to represent secondary ciliary defect but rather to be a distinctive defect only present in PCD patients and widespread in distribution within the ciliary samples. To establish this novel axonemal defect as a primary abnormality, we might achieve the universal and permanent criteria, i.e., to demonstrate the presence of the same abnormality in cilia collected from at least one additional site and at least one additional point in time [26].

Although some genetic defects have been linked to specific ultrastructural ciliary defects, the relationship between clinical findings, genotype and ciliary phenotype is still not clear [27,28]. We did not find any significant difference in the distribution of axonemal asymmetry, mutated gene, clinical findings or ciliary beat pattern in our cohort, but the small number of patients does not allow speculating about a potential correlation.

As previously reported [29], our cohort of PCD patients showed apparently normal ultrastructure in 35% of them, and all of them showed an axonemal symmetry break above 38%. Furthermore, we observed more evident AA in axonemes with an ODA defect, and some of the DCP patients with a higher proportion of ODA defect also showed higher scores of AA. The results of the detailed ultrastructural study that we additionally performed in the ciliary biopsies presumably pointed out the absence of correlation between these two variables. Nevertheless, it also suggested that the AA could indicate the diagnosis in PCD patients with virtually normal axonemal ultrastructure. In this way, the quantitative assessment of the axonemal asymmetry as a new diagnostic tool would enhance the diagnostic yield of TEM.

In this study, we have focused on TEM as a diagnostic procedure of PCD, and we believe that the modified peripheral doublets arrangement detected in our study provides another point of view of the underlying pathogenesis of this condition. Further investigations will be required to validate our findings.

Our study has two main limitations: the small sample size and the potential for observer bias because the axonemal asymmetry analysis is largely based on a subjective description.

In summary, we report a not previously described ciliary ultrastructural defect that could provide a novel diagnostic approach of PCD.

## Figures and Tables

**Table 1:** Summary of Class 1 and Class 2 defects for the ultrastructural diagnosis of PCD (Modified BEAT PCD Criteria).

Class 1 Defects ^1^	Class 2 Defects ^1^
Outer dynein arm defect (>50% cross sections)	Outer dynein arm absence from 25–50% cross sections
Outer and inner dynein arm defect (>50% cross sections)	Combined inner and outer dynein arm absence from 25–50% cross section
	Central complex defect

^1^ Microtubular disorganization defects are excluded.

**Table 2:** Summary of diagnostic tests and CCI of PCD patients according to axonemal asymmetry.

ID	CCI (%)	Genetic Defect	HSVA	TEM Defect	AA (%)
23	40	Not identified	Dyskinetic	No	38
62	60	DNAH9	Dyskinetic	C1	38
74	90	DNAH5	Dyskinetic	C1	40
83	50	DNAH5	Immotile	C1	40
27	40	DNAH5	Immotile	C1	42
44	60	TTC25	Immotile	C1	42
47	70	DNAH5	Immotile	C1	44
55	50	DNAH9	Immotile	No	44
4	40	DNAAF1	Immotile	C1	46
51	60	Not identified	Dyskinetic	No	46
14	60	RSPH1	Dyskinetic	C2	48
32	70	DYX1C1	Immotile	C1	48
80	60	Not identified	Dyskinetic	No	48
91	60	RSPH1	Dyskinetic	No	48
6	50	RSPH1	Normal	C2	50
81	70	DNAH11	Immotile	No	50
22	40	DNAH5	Immotile	C1	56
16	30	Not identified	Immotile	C1	58
92	60	RSPH1	Dyskinetic	No	66
21	60	DNAH5	Immotile	C1	68

Definition of abbreviations: ID = participant identification; CCI = compatible clinical index; HSVA = high-speed video-microscopy analysis; TEM = transmission electron microscopy; C1 = Class 1 defect; C2 = Class 2 defect; AA = axonemal asymmetry.

**Table 3:** Summary of diagnostic tests and CCI of control group according to axonemal asymmetry.

ID	CCI (%)	Genetic Defect	HSVA	TEM Defect	AA (%)
78	30	*	N	No	2
66	40	*	N	No	8
2	20	*	N	No	10
69	50	*	N	No	12
73	30	*	N	No	12
43	20	*	N	No	14
68	50	Not identified	N	No	14
70	40	*	N	No	14
99	30	*	N	No	14
28	30	*	N	No	18
36	20	*	N	No	18
67	40	*	N	No	18
85	40	*	N	No	18
86	40	*	N	No	18
58	40	VUS RSPH4	N	No	22
34	20	*	N	No	24
40	40	*	N	No	34
98	30	*	N	No	36
25	40	*	N	No	44
59	60	VUS CCDC103	N	No	63

Definition of abbreviations: ID = articipant identification; CCI = compatible clinical index; VUS = variant of unknown significance; * = not tested; HSVA = high-speed video-microscopy analysis; TEM = transmission electron microscopy; AA = axonemal asymmetry.

**Table 4:** Distribution of ultrastructural defects with demographic characteristics, ICC, HSVA and axonemal asymmetry.

	Class 1 Defect (n = 19)	Class 2 Defect (n = 5)	No Defect (n = 11)
Age	18.53 (15.9)	22 (21.25)	30.18 (23.17)
Median	15 (8.5, 20)	13 (6, 33)	25 (8.5, 50.5)
Sex			
Female	9 (47.37%)	2 (40%)	7 (63.64%)
Male	10 (52.63%)	3 (60%)	4 (36.36%)
CCI	57.37 (15.93)	56 (15.17)	50 (16.12)
Median	60 (45, 70)	50 (50, 60)	50 (45, 60)
HSVA			
Dyskinetic	5 (26.32%)	2 (50%)	6 (54.55%)
Immotile	13 (68.42%)	1 (25%)	5 (45.45%)
Normal	1 (5.26%)	1 (25%)	0 (0%)
AA	48.27 (9.16)	49.5 (6.61)	50.29 (9.48)
Median	46 (42, 52)	49 (46.5, 52)	48 (47, 54)

Mean (SD)/n (%). Median (1st, 3rd Q.). Definition of abbreviations: CCI = compatible clinical index; HSVA = high-speed video-microscopy analysis; AA = axonemal asymmetry.

**Table 5:** Distribution of HSVA with demographic characteristics, ICC, ultrastructural defects and axonemal asymmetry.

	Dyskinetic (n = 13)	Immotile (n = 19)	Normal (n = 2)
Age	29.23 (23.34)	16.53 (13.55)	23 (24.04)
Median	20 (10, 54)	14 (6, 20)	23 (14.5, 31.5)
Sex			
Female	5 (38.46%)	12 (63.16%)	1 (50%)
Male	8 (61.54%)	7 (36.84%)	1 (50%)
CCI	55.38 (21.06)	54.74 (13.07)	50 (0)
Median	60 (40, 60)	50 (45, 70)	50 (50, 50)
TEM defect			
Class 1	5 (38.46%)	13 (68.42%)	1 (50%)
Class 2	2 (15.38%)	1 (5.26%)	1 (50%)
No defect	6 (46.15%)	5 (26.32%)	0 (0%)
AA	48.55 (10.12)	49.5 (8.7)	49 (1.41)

Mean (SD)/n (%). Median (1st, 3rd Q.). Definition of abbreviations: CCI = compatible clinical index; TEM = transmission electron microscopy; AA = axonemal asymmetry.

## Supplementary Materials

The following are available online at https://www.mdpi.com/article/10.3390/diagnostics12010129/s1, Table S1: Detailed ultrastructural axonemal defects in PCD patients with their axonemal asymmetry proportion, Table S2: Demographic and clinical characteristics of control group, Table S3: Detailed ultrastructural axonemal defects in control group with their axonemal asymmetry proportion, Table S4: Genetics, HSVA and TEM information of PCD patients, Table S5: Demographic and clinical characteristics of PCD patients.
