# Supplementary material for: Axonemal Symmetry Break, a New Ultrastructural Diagnostic Tool for Primary Ciliary Dyskinesia?
Source: Diagnostics (Basel). 2022 Jan 6;12(1):129. doi: 10.3390/diagnostics12010129 (PMC8774754; doi:10.3390/diagnostics12010129)
Supplement: Supplementary file 1 [file diagnostics-12-00129-s001.zip › diagnostics-1507007-supplementary.pdf]

diagnostics

Article

Axonemal Symmetry Break, a New Ultrastructural Diagnostic Tool for Primary Ciliary Dyskinesia?

Rosana Blanco-Máñez [1,*], Miguel Armengot-Carceller [2,3,4,5], Teresa Jaijo [2,6,7] and Francisco Vera-Sempere [8]

[1] Pathology Department, La Fe Polytechnic and University Hospital, 46020 Valencia, Spain
[2] Molecular, Cellularand Genomic Biomedicine Group, IIS La Fe, 46020 Valencia, Spain; miguel.armengot@gmail.com (M.A.-C.); tjaijo@gmail.com (T.J.)
[3] Surgery Department, University of Valencia, 46010 Valencia, Spain
[4] CIBER of Respiratory Diseases (CIBERES), Carlos III Health Institute, Ministerio de Ciencia e Innovación, 28029 Madrid, Spain
[5] ENT Department, La Fe Polytechnic and University Hospital, 46020 Valencia, Spain
[6] Genetics Department, La Fe Polytechnic and University Hospital, 46020 Valencia, Spain
[7] CIBER of Rare Diseases (CIBERES), Carlos III Health Institute, Ministerio de Ciencia e Innovación, 28029 Madrid, Spain
[8] Pathology Department, University of Valencia, 46010 Valencia, Spain; fco.jose.vera@uv.es
* Correspondence: blanco_rosman@gva.es

SUPPLEMENTARY MATERIALS

Table S1. Detailed ultrastructural axonemal defects in PCD patients with their axonemal asymmetry proportion.

ID	Detailed TEM defect (%)[1]	AA (%)
23	CC (10)	38
62	ODA (74)	38
74	ODA (82)	40
83	ODA (98)	40
27	ODA (96)	42
44	ODA (100)	42
47	ODA (96)	44
55	ODA (16)	44
4	ODA (98)+ IDA (62)	46
51	ODA (10)	46
14	CC (32)	48
32	ODA (98)	48
80	ODA (6)	48
91	No defect	48
6	CC (20)	50
81	ODA (4)	50
22	ODA (100)	56
16	ODA (100)	58
92	No defect	66
21	ODA (100)	68

[1] Expressed as a percentage of affected ciliary sections.
Definition of abbreviations: ID= participant identification; TEM= Transmission Electron microscopy; CC= Central complex defect; ODA= Outer dynein arm defect; IDA= Inner dynein arm defect; AA= Axonemal asymmetry.

Table S2. Demographic and clinical characteristics of control group (n=20).

	n	%
**Male/Female**	**11/9**	**55/45**
**Age (years)**	**16,4(5-52)**	
**Clinical characteristics**		
NRD	6	30
Rhinorrhea	13	65
Cronic wet cough	15	75
Pneumonia	10	50
Bronchiectasis	7	35
Laterality defects	2	10
Fertility problems[1]	1	20

Definition of abbreviations: NRD= Neonatal respiratory distress. [1] In patiens older than 18 years.

Table S3. Detailed ultrastructural axonemal defects in control group with their axonemal asymmetry proportion.

ID	Detailed TEM defect (%)[1]	AA (%)
78	No defect	2
66	No defect	8
2	No defect	10
69	ODA (4)	12
73	ODA (8)	12
43	ODA (2)	14
68	ODA (2)	14
70	ODA (4)	14
99	No defect	14
28	No defect	18
36	No defect	18
67	ODA (8)	18
85	No defect	18
86	ODA (2)	18
58	No defect	22
34	No defect	24
40	ODA (2)	34
98	ODA (6)	36
25	ODA (22)	44
59	ODA (16)	63

[1] Expressed as a percentage of affected ciliary sections. Definition of abbreviations: ID= participant identification; TEM= Transmission Electron microscopy; ODA= Outer dynein arm defect; AA= Axonemal asymmetry.

Table S4. Genetics, HSVA and TEM information of PCD patients (n=20).

	n	%
**Ultrastructural defect**		
C1	11	55
C2	2	10
No defect	7	35
**Mutated gene**		
DNAH5	6	30
DNAH9	2	10
RSPH1	4	20
TTC25	1	5
DNAAF1	1	5
DYX1C1	1	5
DNAH11	1	5
Not identified	4	20
**HSVA**		
Dyskinetic	8	40
Immotile	11	55
Normal	1	5

Definition of abbreviations: ID= Participant identification; C1= Class 1 defect; C2= Class 2 defect; HSVA= High speed videomicroscopy analysis.

Table S5. Demographic and clinical characteristics of PCD patients (n=20).

	n	%
**Male/Female**	11/9	55/45
**Age (years)**	24 (1-64)	
**Clinical characteristics**		
NRD	13	65
Rhinorrhea	17	85
Cronic wet cough	18	90
Pneumonia	10	50
Bronchiectasis	13	65
Laterality defects	8	40
Fertility problems[1]	3	33,3

Definition of abbreviations: NRD= Neonatal respiratory distress. [1] In patiens older than 18 years.
